# Progress Toward Poliomyelitis Eradication — Pakistan, January 2020–July 2021

**DOI:** 10.15585/mmwr.mm7039a1

**Published:** 2021-10-01

**Authors:** Chukwuma Mbaeyi, Shahzad Baig, Zainul Khan, Hamish Young, Millhia Kader, Jaume Jorba, Muhammad Rana Safdar, Hamid Jafari, Richard Franka

**Affiliations:** ^1^Global Immunization Division, Center for Global Health, CDC; ^2^National Emergency Operation Center, Islamabad, Pakistan; ^3^World Health Organization, Islamabad, Pakistan; ^4^UNICEF, Islamabad, Pakistan; ^5^Division of Viral Diseases, National Center for Immunization and Respiratory Diseases, CDC; ^6^Expanded Program on Immunization, Ministry of National Health Services Regulation and Coordination, Islamabad, Pakistan; ^7^World Health Organization, Amman, Jordan.

When the Global Polio Eradication Initiative began in 1988, wild poliovirus (WPV) transmission was occurring in 125 countries; currently, only WPV type 1 (WPV1) transmission continues, and as of August 2021, WPV1 transmission persists in only two countries (*1*,*2*). This report describes Pakistan’s progress toward polio eradication during January 2020–July 2021 and updates previous reports (*3*,*4*). In 2020, Pakistan reported 84 WPV1 cases, a 43% reduction from 2019; as of August 25, 2021, Pakistan has reported one WPV1 case in 2021. Circulating vaccine-derived poliovirus (cVDPV) emerges as a result of attenuated oral poliovirus vaccine (OPV) virus regaining neurovirulence after prolonged circulation in underimmunized populations and can lead to paralysis. In 2019, 22 cases of cVDPV type 2 (cVDPV2) were reported in Pakistan, 135 cases were reported in 2020, and eight cases have been reported as of August 25, 2021. Because of the COVID-19 pandemic, planned supplementary immunization activities (SIAs)* were suspended during mid-March–June 2020 (*3*,*5*). Seven SIAs were implemented during July 2020–July 2021 without substantial decreases in SIA quality. Improving the quality of polio SIAs, vaccinating immigrants from Afghanistan, and implementing changes to enhance program accountability and performance would help the Pakistan polio program achieve its goal of interrupting WPV1 transmission by the end of 2022.

## Immunization Activities

**Essential (routine) immunization.** For 2020, the World Health Organization (WHO) and UNICEF estimated Pakistan’s national coverage with 3 doses of OPV and 1 dose of inactivated poliovirus vaccine by age 12 months at 83% and 85%, respectively (*6*). A 2021 survey sponsored by WHO and Gavi, the Vaccine Alliance, indicated that the proportion of fully immunized children aged 12–23 months, by province, ranged from 37.5% in Balochistan to 88.9% in Punjab. None of the districts in the provinces of Balochistan, Khyber Pakhtunkhwa, and Sindh achieved ≥80% coverage among children aged 12–23 months, including the core WPV1 reservoir districts (i.e., districts with persistent, intractable poliovirus circulation) in Quetta (45.5% coverage), Peshawar (76.6%), and Karachi (63.9%). In comparison, 31 of 36 (86%) districts in Punjab province achieved ≥80% coverage.

**Supplementary immunization activities.** Pakistan was among 155 OPV-using countries that ceased all use of OPV type 2 in 2016; the standard product for outbreak response to confirmed cVDPV2 outbreaks is monovalent OPV type 2 (mOPV2; containing Sabin-strain type 2) (*7*). The Global Polio Eradication Initiative authorized restarting the filling of stocks of trivalent OPV (tOPV; containing Sabin-strain types 1, 2, and 3) for programs to use in SIAs where WPV1 and cVDPV2 cocirculate, for efficiency in scheduling and implementation. During 2020, four national immunization days (NIDs) and two subnational immunization days (SNIDs) targeting children aged <5 years were conducted using bivalent OPV (bOPV; containing Sabin-strain types 1 and 3) and, in areas with cVDPV2 transmission, either mOPV2 or tOPV. Suspension of SIAs during March–June 2020 was related to control measures for the COVID-19 pandemic, including procurement of personal protective equipment for vaccination teams. SIAs resumed in July 2020 with a small-scale mOPV2 case-response vaccination campaign, followed by a broader mOPV2 SNID in August, a tOPV SNID in October, and bOPV NIDs in September and November 2020.

The overall percentage of missed children who were identified (i.e., targeted children who were not vaccinated during SIAs) in 2020 increased from 1.2% during the February 2020 NIDs to 1.8% during the NIDs in September 2020. Although the proportion of missed children has remained low nationwide, substantial gaps in identifying missed children persist at the subnational level, especially in the core WPV1 reservoirs, with several districts reporting >5% of children aged <5 years missed during NIDs. Collectively, hundreds of thousands of children are repeatedly being missed among approximately 40 million children targeted during each NID. Lot quality assurance sampling (LQAS) survey^†^ results have indicated performance gaps at union councils (subdistricts) identified to be at highest risk for poliovirus transmission in 2020, with 12%–43% of these union councils’ SIAs failing to achieve the 90% LQAS pass threshold.

In 2021, two NIDs have been conducted to date: one using tOPV in January and another in March using bOPV or tOPV, depending upon the area. Combined bOPV and tOPV SNIDs were conducted in June and August; bOPV NIDs are planned for September and December. Smaller, targeted case-response vaccination activities have also been completed during 2020–2021. Approximately 1.4% of 40 million children targeted were reported as missed following the January 2021 NIDs, including 471,743 who were not available at the time of the campaigns and 125,087 whose caregivers refused to have their eligible children vaccinated.

## Poliovirus Surveillance

**Acute flaccid paralysis surveillance.** Pakistan reported a national nonpolio acute flaccid paralysis (AFP)^§^ rate of 15.3 cases per 100,000 persons aged <15 years in 2020 (Table); provincial rates ranged from 8.8 to 15.6. As of June 27, 2021, the annualized 2021 nonpolio AFP rate is 10.3, and stool adequacy^¶^ rates during 2020 and 2021 exceeded ≥80% nationally and in each province.

**TABLE:** Acute flaccid paralysis surveillance indicators, number of wild poliovirus cases reported, and number of circulating vaccine-derived poliovirus type 2 cases reported, by region and period — Pakistan, January 2020–July 2021

Region	AFP surveillance indicators				Poliovirus cases							
	No. of AFP cases (nonpolio AFP rate*)		% with adequate stool specimens^†^		Reported WPV1 cases				Reported cVDPV2 cases			
	2020	2021^§^	2020	2021	Jan–Jun 2020	Jul–Dec 2020	Jan–Jun 2021	Total	Jan–Jun 2020	Jul–Dec 2020	Jan–Jun 2021	Total
Azad Jammu and Kashmir	212 (11.3)	91 (9.9)	90.1	93.4	0	0	0	0	0	0	0	0
Gilgit-Baltistan	106 (15.6)	58 (17.2)	85.9	81.0	0	0	0	0	0	0	0	0
Islamabad	120 (12.0)	62 (12.7)	85.0	88.7	0	0	0	0	0	0	0	0
Khyber Pakhtunkhwa	2,732 (15.4)	1,212 (11.5)	82.3	85.3	21	1	0	22	42	0	1	43
Punjab	5,744 (11.1)	2,415 (9.6)	84.9	87.7	4	10	0	14	6	19	1	26
Balochistan	547 (8.8)	248 (8.5)	84.8	90.7	15	11	1	27	1	22	4	27
Sindh	2,511 (11.0)	975 (8.8)	88.6	92.1	20	2	0	22	3	42	2	47
**Total**	**11,972 (15.3)**	**5,061 (10.3)**	**85.4**	**88.3**	**60**	**24**	**1**	**85**	**52**	**83**	**8**	**143**

**Abbreviations**: AFP = acute flaccid paralysis; cVDPV2 = circulating vaccine-derived poliovirus type 2; WHO = World Health Organization; WPV1 = wild poliovirus type 1.

* Nonpolio AFP cases per 100,000 persons aged <15 years.

^†^ Defined as two stool specimens collected ≥24 hours apart within 14 days of paralysis onset and arriving at a WHO-accredited laboratory with reverse cold chain maintained and without leakage or desiccation.

^§^ Annualized.

**Environmental surveillance.** Routine sewage sampling at designated sites augments AFP surveillance to enhance timely detection of poliovirus circulation. Pakistan has 68 environmental surveillance sampling sites. During 2020, among 768 tested sewage samples, 53% (407) tested positive for WPV1 compared with 47% of 786 samples tested in 2019. In 2021, to date, 12% (61) of 513 samples have tested positive for WPV1 compared with 55% of 566 samples during the same period in 2020. The geographic distribution of positive samples and the detection of orphan viruses (those that are ≥1.5% divergent from their closest genetic match, indicating gaps in AFP surveillance sensitivity) across several provinces indicate persistent widespread circulation of WPV1 outside the core reservoirs. Further, 136 sewage samples (18%) were positive for cVDPV2 in 2020, compared with 40 (5%) in 2019, and 32 (6%) in 2021 to date.

**FIGURE 1:**
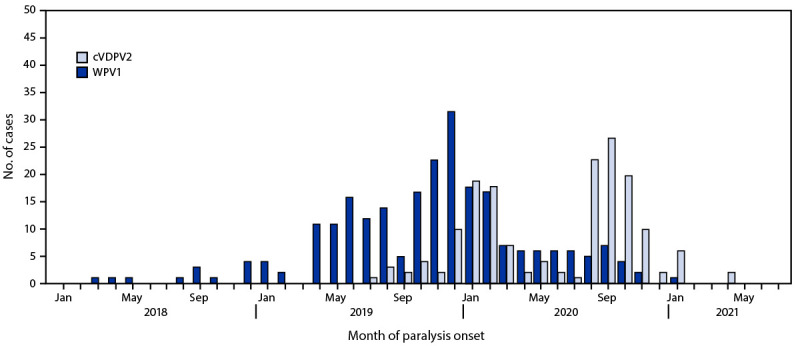
Wild poliovirus type 1 and circulating vaccine-derived poliovirus type 2 cases, by month — Pakistan, January 2018–July 2021 **Abbreviations:** cVDPV2 = circulating vaccine-derived poliovirus type 2; WPV1 = wild poliovirus type 1.

**FIGURE 2:**
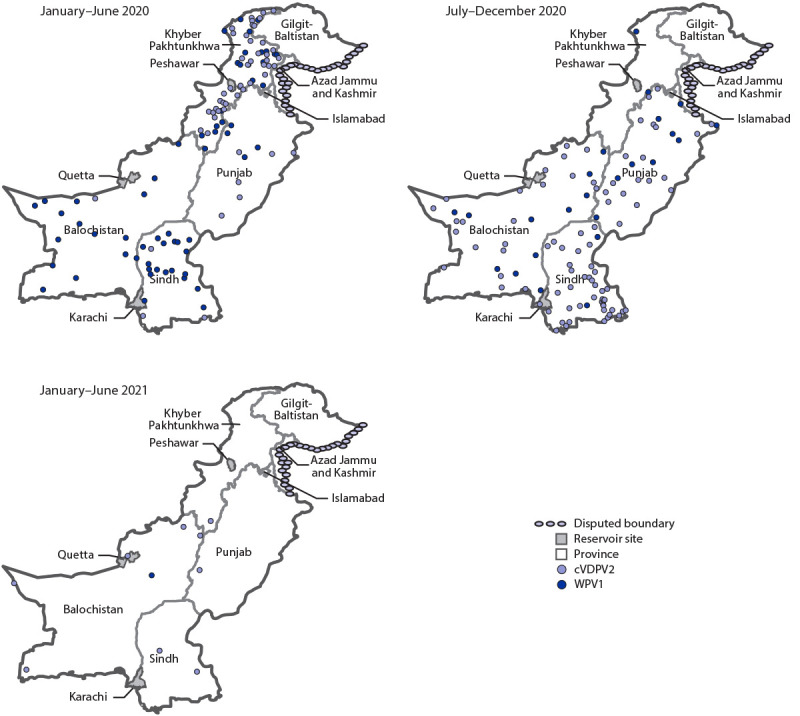
Location of cases of wild poliovirus type 1 and circulating vaccine-derived poliovirus type 2, by province and period — Pakistan, January 2020–June 2021 **Abbreviations:** cVDPV2 = circulating vaccine-derived poliovirus type 2; WPV1 = wild poliovirus type 1.

**Epidemiology of poliovirus cases.** During 2020, 84 WPV1 cases were reported in Pakistan, a 43% reduction from the 147 cases reported in 2019. As of August 25, 2021, a single WPV1 case (Killa Abdullah, Balochistan province) has been reported in 2021, compared with 71 cases from 33 districts during the same period in 2020. Among the 85 cases reported during January 2020–July 2021 (Figure 1), 27 (32%) were in Balochistan, 22 (26%) in Sindh, 22 (26%) in Khyber Pakhtunkhwa, and 14 (16%) in Punjab (Figure 2).

The WPV1 patients’ ages ranged from 3 months to 13 years (median = 18 months); 58% had never received OPV, 19% had received 1–2 doses through essential immunization, and 23% had received ≥3 OPV doses. Genetic analysis indicated that seven clusters (groups of polioviruses sharing ≥95% sequence identity in the region coding the VP1 capsid protein) were identified from WPV1 cases and environmental surveillance isolates during January 2020–June 2021; only two of these clusters have been detected in 2021 to date.

Ongoing cVDPV2 transmission from several emergences in Pakistan has resulted in 165 cVDPV2 cases during July 2019–July 2021 (22 cases in 2019, 135 in 2020, and eight in 2021 to date), with the most recent case onset on April 23, 2021 (Figure 1). Of the 165 cVDPV2 cases, 59 (36%) were in Khyber Pakhtunkhwa, 47 (29%) in Sindh, 27 cases (16%) each in Punjab and Balochistan, four (2%) in Gilgit-Baltistan, and one (1%) in Islamabad (Figure 2). The ages of the children with cVDPV2 cases ranged from 2 months to 12 years (median = 18 months).

## Discussion

After a series of setbacks in 2019, fewer WPV1 cases have been reported in Pakistan during 2020–2021 to date, with a concomitant reduction in the proportion of WPV1-positive environmental surveillance samples. These findings are associated with implementation of planned improvements in program management and accountability (*8*) that began before the COVID-19 pandemic and have continued during the pandemic. In contrast, the cVDPV2 outbreak that began in July 2019 intensified in 2020. Transmission of cVDPV2 has decreased considerably in 2021 after large-scale, type 2–containing OPV SIAs.

Although the number of WPV1 cases declined substantially during 2020, the geographic distribution of cases, continued isolation of orphan viruses in sewage samples, and persistent WPV1 circulation in the core reservoirs could signal that efforts to interrupt the circulation of polioviruses in Pakistan are in jeopardy as the high transmission season in the last quarter of the year approaches. Notably, the observed changes in poliovirus detections occurred in the face of the challenges that the COVID-19 pandemic posed to effective immunization activities, but these observations might also be related to a decrease in community interactions during the pandemic.

Despite meeting critical AFP surveillance indicator benchmarks at the national and provincial levels in 2020, the overall number of AFP cases detected declined by approximately 20% from 2019, coincident with the disruption of active AFP surveillance activities and field investigations and repurposing of personnel and resources in response to the COVID-19 pandemic (*3*). However, environmental surveillance findings suggest levels of poliovirus circulation declined during the reporting period.

Recurrent challenges with vaccination campaign quality could undercut efforts to interrupt virus transmission. To achieve the dual goals of eliminating WPV1 and halting cVDPV2 transmission, efforts are warranted to increase the quality of polio SIAs by further decreasing the number of children who were repeatedly missed in the WPV1 reservoirs. In light of the increasing political instability in Afghanistan, enhanced efforts and contingency plans are critical to ensure vaccination of children of families migrating into Pakistan.

To increase vaccine acceptance and community engagement, the Pakistan polio program should consider focusing ancillary initiatives (e.g., integrated health and clean water service delivery) on the highest priority areas and tailor them to the perceived needs of communities (*9*). Accelerated implementation of the proposed program transformation would improve management and accountability at all levels of the program. This includes prioritizing the recruitment and training of frontline workers who are empowered to provide culturally relevant leadership that is accepted by the local communities. The program must act with urgency to take advantage of the opportunity presented by the slowing of poliovirus circulation in 2021 to eliminate all virus transmission from the country by the end of 2022 (*9*,*10*).

**Summary**

**What is already known about this topic?**

Pakistan is one of two countries (including Afghanistan) where wild poliovirus type 1 (WPV1) transmission has never been interrupted.

**What is added by this report?**

WPV1 cases in Pakistan declined by 43% from 2019 to 2020, and only one case has been reported to date in 2021. A circulating vaccine-derived poliovirus type 2 (cVDPV2) outbreak that began in 2019 has slowed substantially in 2021 following implementation of large-scale type 2–containing oral poliovirus vaccination campaigns.

**What are the implications for public health practice?**

For WPV1 and cVDPV2 transmission to be eliminated, efforts are warranted by the Pakistan polio program to reduce the number of persistently missed children and ensure vaccination of children migrating into Pakistan because of political instability in Afghanistan.

## Footnotes

* SIAs are mass house-to-house vaccination campaigns targeting children aged <5 years with OPV, regardless of the child’s vaccination history.

† LQAS uses a small sample size to assess the quality of vaccination activities after SIAs in union councils (referred to as “lots”). LQAS surveys seek evidence of vaccination (finger marking) by random selection of 60 children within each lot. If the number of unvaccinated persons in the sample exceeds three, then the union council SIA is classified as having failed at a threshold of ≥90%, and additional vaccination activities in those areas are recommended. If the threshold of ≥90% (three or fewer unvaccinated children) is met, the union council SIA is classified as having passed.

§ AFP cases that are discarded as not having laboratory or other proof of poliovirus as the cause are called nonpolio AFP cases. The expected background rate of nonpolio AFP illnesses is ≥2 per 100,000 children aged <15 years per year, the standard WHO performance indicator target for sufficiently sensitive detection. The standard WHO stool specimen indicator target is adequate stool specimen collection from ≥80% of AFP cases.

¶ Stool specimens are considered adequate if two specimens are collected ≥24 hours apart within 14 days of paralysis onset and arrive at a WHO-accredited laboratory with reverse cold chain maintained and without leakage or desiccation.
